# Whole Body Vibration Training - Improving Balance Control and Muscle Endurance

**DOI:** 10.1371/journal.pone.0089905

**Published:** 2014-02-26

**Authors:** Ramona Ritzmann, Andreas Kramer, Sascha Bernhardt, Albert Gollhofer

**Affiliations:** 1 Institute of Sport and Sport Science, University of Freiburg, Freiburg, Germany; 2 Department of Sports Sciences, University of Konstanz, Konstanz, Germany; University of Rome Foro Italico, Italy

## Abstract

Exercise combined with whole body vibration (WBV) is becoming increasingly popular, although additional effects of WBV in comparison to conventional exercises are still discussed controversially in literature. Heterogeneous findings are attributed to large differences in the training designs between WBV and “control” groups in regard to training volume, load and type. In order to separate the additional effects of WBV from the overall adaptations due to the intervention, in this study, a four-week WBV training setup was compared to a matched intervention program with identical training parameters in both training settings except for the exposure to WBV. In a repeated-measures matched-subject design, 38 participants were assigned to either the WBV group (VIB) or the equivalent training group (CON). Training duration, number of sets, rest periods and task-specific instructions were matched between the groups. Balance, jump height and local static muscle endurance were assessed before and after the training period. The statistical analysis revealed significant interaction effects of group×time for balance and local static muscle endurance (p<0.05). Hence, WBV caused an additional effect on balance control (pre vs. post VIB +13%, p<0.05 and CON +6%, p = 0.33) and local static muscle endurance (pre vs. post VIB +36%, p<0.05 and CON +11%, p = 0.49). The effect on jump height remained insignificant (pre vs. post VIB +3%, p = 0.25 and CON ±0%, p = 0.82). This study provides evidence for the additional effects of WBV above conventional exercise alone. As far as balance and muscle endurance of the lower leg are concerned, a training program that includes WBV can provide supplementary benefits in young and well-trained adults compared to an equivalent program that does not include WBV.

## Introduction

Whole-body vibration (WBV) is a training method that exposes the entire body to mechanical oscillations while standing on a vibrating platform [1]. Although WBV combined with resistance training is becoming increasingly popular in various training recommendations, there is still an ongoing debate in literature, whether there are additional effects of WBV on muscle fitness and muscle performance in comparison to conventional exercise [2]. On one hand, there is evidence for an additive effect of WBV beyond that of conventional exercises regarding strength gains [3], [4], power [5], [6], flexibility [7], [8] and adaptations in motor control [9], [10]. On the other hand, there are several other studies, in which WBV-induced performance improvements could not be found [11]–[13], and some authors question if there are any additional effects of WBV if the training protocols of workouts with and without exposure to WBV are matched [14]. In the existing literature, these controversial observations can be allocated to the following three aspects [14]:

1. Divergence in WBV Training protocols: There are incompatibilities in WBV training regarding experimental protocols and inconsistently adjusted WBV parameters in WBV intervention studies. Hence, functional responses and training adaptations are hardly comparable between studies [1]. WBV training setups are characterized mainly by the adjustment of WBV parameters such as vibration frequency, amplitude and direction; the latter being defined by the vibration type [2]. Each of these WBV factors is known to significantly influence training adaptation and modulates WBV-induced physiological responses [1]. For instance, changes in energy metabolism and turnover [15], blood flow [16]–[18] and neuromuscular activation [19]–[21] have been shown to be highly dependent on the specific parameter selection. Furthermore, these changes were accompanied by a variety of functional responses [1], [2] like different effects on muscle power [22]and vertical jump height [23].

2. Matched Controls: Large differences in training designs as well as variations in training load, volume or type between WBV and control group training assumably are the main cause for the inhomogeneous results of these intervention studies [14], [1]. The activities of the control groups vary remarkably from training settings different to that of the treatment group [10], [24], [25], [5] up to control groups not performing any extra exercise at all [3], [26]. There are only a few studies, in which both groups performed identical exercises [13], [11], [4], [12]. Among those studies, Kvorning et al. [12], Ronnestaad et al. [13] and De Ruiter et al. [11] could not find any additional effects in strength and jump performance caused by WBV exposure compared to the control groups.

3. Intervention Design: There is very little scientific evidence about the optimal duration for WBV exposure [1]. Usually, WBV training protocols are characterized by a short duration of 1–5 min of intermittent or continuous WBV application (for review see [2]). Particularly when a high demand on the neuromuscular system accompanied by metabolic stress is required also exhausting protocols have been taken into account [1].

Hence, based on the three aspects mentioned above, the potential benefits of WBV in comparison to traditional forms of exercise still remain unclear. Despite the substantial amount of vibration-related articles there is a lack of controlled training intervention studies providing equivalent training settings for WBV and control groups regarding training load, volume and type. Disregarding these methodological constraints, there is general evidence in literature that WBV training causes a variety of adaptations associated with performance improvements [2]. For instance, enhancements in energy metabolism [27], muscle perfusion [18] and peripheral circulation [28] point towards distinct alterations in endurance capacities, although the WBV-induced effects on endurance per se has not yet been studied. It has also been demonstrated that force generating capacities [29], [23] are increased after WBV training and that these adaptations may improve jump performance; accordingly, strength gains [4], [29], [23] and enhanced motor capabilities [30], [10] are also associated with improvements in balance control. Moreover, one can expect that the vibration stimulus, which is associated with improvements in neuromuscular control and an augmented in joint stiffness [31], [32], could enhance postural control [5], [33] due to improved ankle [34] and knee joint stability [35], [36].

This study aimed to investigate whether a WBV intervention program results in superimposed improvements in balance control, jump performance and local static muscle endurance in comparison to an equivalent intervention program performed without vibration. Balance control, jump performance and muscle endurance were selected based on already existing fundamental studies providing WBV specific adaptation in metabolic power [37], [38], energy expenditure [15], [37], strength [29], [4] and motor control [30], [10]. We hypothesized that four weeks of additional WBV training would induce superimposed adaptations in the selected criteria in well-trained healthy adults.

## Methods

### Experimental Design

A repeated-measures matched-subject design was used to evaluate the effect of a four-week WBV training regimen versus an equivalent control training. In order to separate the additional effect of WBV from the overall adaptations due to the intervention, training settings for the VIB and the CON group were matched regarding training load, volume and type. Thus, the VIB group performed the same exercise as the CON group, except for an additional exposure to WBV.

Three protocols were used to assess performance adaptations in response to training interventions: In protocol 1 we investigated the effect of both training regimes on balance control in a postural task. Protocol 2 assessed changes in jump performance and protocol 3 quantified alterations in local static muscle endurance.

### Subjects

Forty subjects (17 females, 23 males, age 25±4) with no history of neurological disorders or injuries to the lower extremities volunteered to participate in this study. The volunteers were physically fit students in the department of sports science, had not used WBV training before and regularly participated in sports activities containing jumping and running (volleyball, basketball, track and field, etc.) to minimize variations in jumping performance and to guarantee high fatigue resistance during the measurements. The subjects were encouraged to maintain their dietary, sleeping, drinking and activity habits during the participation in the study.

All subjects gave written informed consent to the experimental procedure, which was approved by the ethics committee of the University of Freiburg (Nr. 132/09) and was in accordance with the latest revision of the Declaration of Helsinki and with [22]. The subjects were assigned to either the WBV training group (VIB, 9 female and 11 male participants, age 25±4 years, height 172±12 cm and weight 72±14 kg) or the control training group (CON, 8 female and 12 male participants, age 25±3 years, height 173±09 cm and weight 69±11 kg). Prior to the experiment, the sample size was estimated by means of a power analysis (f = 0.25; alpha = 0.05; Power = 0.85).

Two male participants dropped out because of an ankle joint injury and illness, respectively.

### Procedures

#### Training

**Figure 1:**
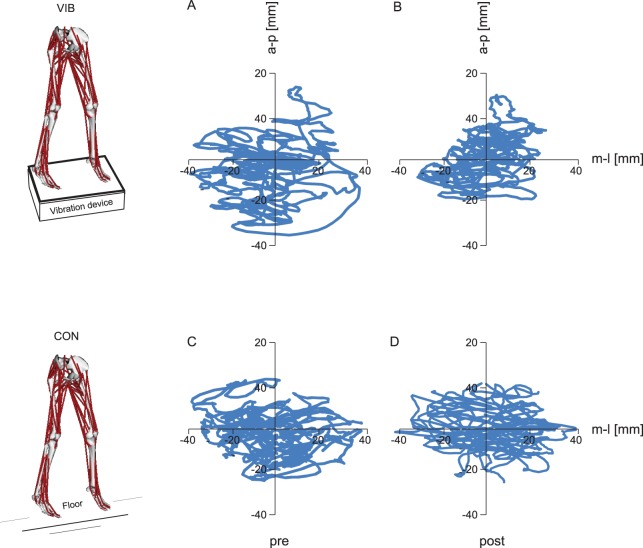
Reduction in COP displacement in two representative subjects of the VIB and CON group. Centre of pressure (COP) displacement of one subject of the whole body vibration (VIB) group (A and B) and one subject of the equivalent training (CON) group (C and D) in anterior-posterior (a–p) and medio-lateral (m-l) direction before and after the training interventions. The participants of both training groups trained in an upright standing position with a knee flexion of 10° in the forefoot stance.

All participants trained for a period of four weeks with three training sessions per week and at least one day of rest between two sessions. All sessions were documented, surveyed, and supervised by the authors of the study. In order to separate the WBV effects from the overall adaptations due to plain intervention, the training of the CON group was matched with the training of the WBV group, i.e. training duration, number of sets, rest periods and task-specific instructions were identical for both groups (Table 1). For all subjects, one training session consisted of two sets. The duration of the first set was established at 3 min in the first week, 5 min in the second and 8 min in the third and fourth week, respectively. For the second set the subjects trained until exhaustion, however, duration was limited to 20 min. A subject was considered to be exhausted when it was impossible for him or her to stand on the vibration device. The sets were separated by a 3 min rest period with the subjects sitting on a chair. During training the subjects of both groups maintained an upright body position, stood on their forefoot with their heels off the ground and the knee joints flexed at an angle of 10° (Fig. 1). They placed their hands on their hips, directed their head and eyes forward and distributed their weight equally on both feet with a foot-to-foot distance of 42 cm.

**Table 1:** Means and standard deviations of training duration in the CON and VIB training group.

Group	Sets	Duration week 1		Duration week 2		Duration week 3		Duration week 4	
		Set 1	Set 2	Set 1	Set 2	Set 1	Set 2	Set 1	Set 2
VIB	2	3′	10′15″±1′8″	5′	13′14″ ±2′4″	8′	14′27″ ±1′29″	8′	14′51″ ±58″
CON	2	3′	9′49″ ±1′27″	5′	13′40″ ±1′57″	8′	14′59″ ±1′52″	8′	14′46″ ±1′33″

The CON training was performed on the floor whereas the VIB training was performed on a side alternating vibration platform (Galileo Sport, Novotec Medical, Pforzheim, Germany), which generated vibration by platform oscillations along the sagittal axis. The vibration frequency was set to 25 Hz and the amplitude to 4 mm. The WBV training set-up was designed in accordance to recently given WBV recommendations [21], [20], [19], because this exact setting has been shown to provide high activation intensities in the leg muscles and therefore particularly high neuromuscular demands during vibration training. All participants were thoroughly introduced to WBV training by the authors.

#### Outcome measures

A pre and post data assessment was conducted at least two days before and two days after the training period. Prior to data collection, participants underwent a 10-min warm-up phase consisting of running, tapping and hopping. The three protocols were performed on the same day in the same order (1-2-3).

To provide comparable baseline values in both groups, every subject in the CON was matched evenly to an equivalent in the VIB group along the variables of the three protocols. Thus, prior to training each of the participants in the CON group was assigned to a participant in the VIB group based on equivalent baseline values so that the groups had the same initial level. This pairing was also used to match the training duration: whereas the duration of the first training set was defined, the duration of the second set for each of the CON subjects was matched to the duration performed by the assigned participants in the VIB group.

#### Protocol 1 - Balance control

In order to quantify balance control, the subjects performed a one-legged balance task on a spinning top (40 cm in diameter and a height of 9 cm), which was positioned on a force plate (AMTI, Watertown, USA). All participants performed the balance task on their preferred leg, barefoot, knees extended, eyes opened and hands akimbo [39]. During each of the measurements the subjects were instructed and supervised by the authors. The participants performed three trials with durations of 30 seconds each and a rest period of one minute between each trial. Prior to the actual experiment, each subject performed three test trials on the spinning top to get accustomed to the unstable surface and to reduce the impact of learning effects on the study.

Balance performance was quantified by means of the COP movement. For each subject and trial, the COP displacement was recorded with a frequency of 40 Hz and the overall COP displacement in medial–lateral (COP_ml_) and anterior–posterior (COP_ap_) direction for every subject was assessed and averaged for his or her three recorded trials. Additionally, total COP movement (COP_t_) was calculated according to the Pythagoras theorem by means of the formula COP(t) = Σ D_i_, i = [0;1200] with D_i_ = [(Displacement in anterior-posterior axis)^2^+(Displacement in medio-laterlal)^2^]^½^ for each sample point [40], [24]. The mean values of the three trials were used for statistical analysis.

#### Protocol 2- Jump performance

Vertical counter-movement jumps (CMJ) were used to assess jump performance. During CMJ, the angular displacement of the knees was standardized so that the subjects were required to bend their knees to approximately 90°. Horizontal and lateral displacements were minimized, and the hands were kept on the hips throughout the jump. Three barefoot maximal jumps were performed on a force plate (Leonardo, Novotec Medical GmbH, Pforzheim, Germany) with one minute rest between trials. Prior to the actual experiment, each subject performed three jumps for familiarization. The flight time (t) of each single jump was calculated and used to determine the increase in the center of gravity above the ground *h* (height in m), i.e., *h* = *gt* ^2^/8, where *g* = 9.81 [40], [24]. Flight time was defined as the time interval from the instant of take-off to the instant of landing. The threshold for take-off and landing was set to 3 Newton. The mean value of the three jumps was used for statistical analysis.

#### Protocol 3 - Local static muscle endurance

Muscle endurance was assessed in a static endurance task. The subjects were standing on the forefoot (with the heels in 4,5 cm distance to the floor) with their knees extended and a weight equal to 60% of their body weight placed on their shoulders. To control for the ankle joint position a foam cube was used. This cube (4 cm high) was fixed under the subjects’ left and right heel [21]. The additional load was applied via a standard weightlifting bar (180 cm in length, attached to a frame to avoid body and trunk movement) with weight plates attached to each side. The bar was surrounded by foam for comfort and positioned on the subject’s shoulders.

Local static muscle endurance was estimated by means of the time interval from the beginning of lifting the heel until the instant where the subjects could not hold their heels in a distance of 4 cm from the floor anymore.

Prior to this experiment, each subject performed a short test trial of 10 seconds.

### Statistical Analyses

The statistical analyses were executed using SPSS 17.0 (SPSS Inc., Chicago, Illinois). The effect of the training programs on the three variables balance control, jump performance and local static muscle endurance was evaluated using a two-factor analysis of variance, group [2, VIB vs. CON]×time [2, pre vs. post]. Apriori, Mauchly’s test was used to test for sphericity; the variances of the differences between all combinations of the groups were equal. The normality of the data was evaluated with Kolmogorov-Smirnov; data followed a normal distribution. In case the ANOVA revealed significant changes (P<0.05), the differences between values before and after training were compared by a one-tailed student’s *t*-test which was corrected by Bonferroni adjustments. The level of significance was set to p≤0.05 and statistically significant differences were marked with a symbol (*).

Group data are presented as means ± standard deviations (SD) unless stated otherwise.

## Results

Significant interaction effects were observed for group×time for balance and local static muscle endurance (P<0.05). No interaction effects were observed regarding jump height (P = 0.16).

### 1. Balance

**Figure 2:**
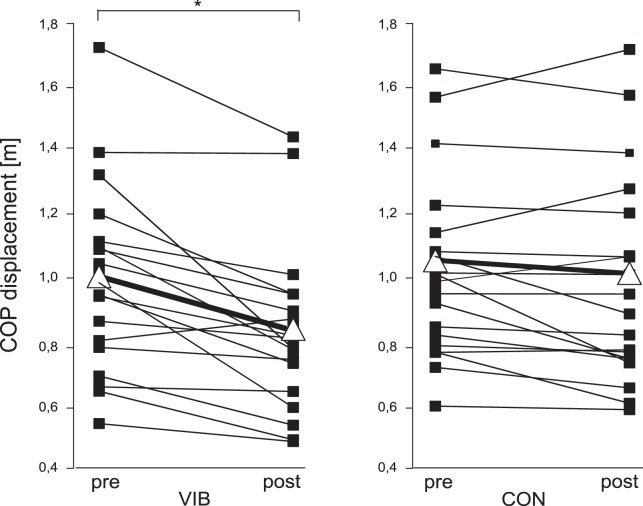
Group mean data of balance performance. Mean centre of pressure (COP) displacement over three trials before (pre) and after (post) the intervention (marked as ▪) from each single subject in the whole body vibration training group (VIB) and the equivalent training group (CON) as well as the mean of all subjects (marked as Δ). Values obtained after training are expressed as percentages of the baseline value. The CON group showed no changes, whereas in VIB group the COP displacement was significantly reduced.

Figure 1A and 1B show the COP movements of a single subject before and after the WBV training. The COP movements of a single subject of the CON group before and after the intervention are illustrated in figures 1C and 1D. The analysis of variance revealed a significant interaction effect of group×time. For instance, the COP displacement decreased significantly more for the VIB than for the CON group. Moreover, a significant main effect for time was observed (P<0.05), indicating a decreased sway path after the training period for both groups. The VIB group significantly reduced COP displacement in response to training (COP_t_ –13% (p<0.05), COP_ml_ −16% (p<0.05) and COP_ap_ −15% (p<0.05)). Changes in the CON group remained insignificant (COP_t_ −6% (p = 0.33), COP_ml_ −9% (p = 0.25) and COP_ap_ −5% (p = 0.98)). The grand means of COP movements and changes for each single subject of the VIB and CON group are illustrated in Fig. 2.

### 2. Jump Performance

**Figure 3:**
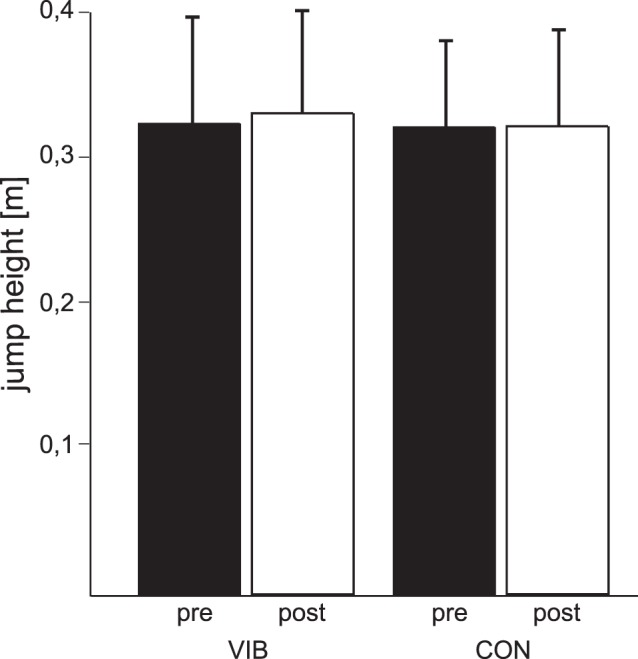
Group mean data of jump height. Grand mean of the jump height for the whole body vibration training group (VIB) and the equivalent training group (CON) before (white) and after (black) the intervention. Values obtained after the training are expressed as percentages of the baseline values. Neither the CON group nor the VIB group showed significant changes in response to training.

The ANOVA revealed no significant differences for the factors group and time. The grand means of jump height for the VIB and the CON groups are illustrated in Figure 3. Neither the VIB group (+2.5%, p = 0.25) nor the CON group (+0.5%, p = 0.82) showed changes in response to the training regimens.

### 3. Local Static Muscle Endurance

**Figure 4:**
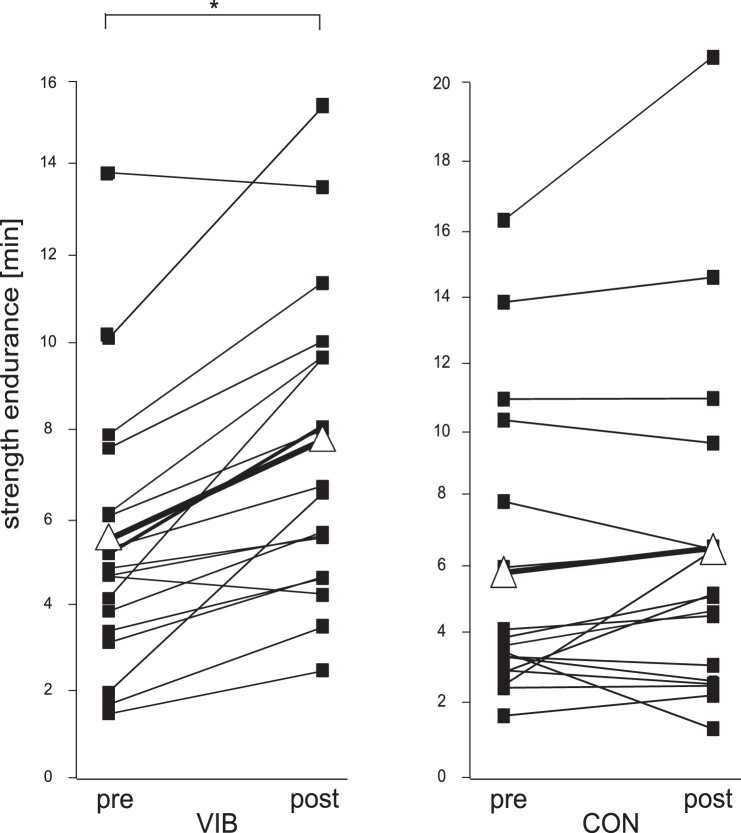
Group mean data of muscle endurance. Muscle endurance before (pre) and after (post) the intervention (marked as ▪) from each single subject in the whole body vibration training group (VIB) and the equivalent training group (CON) and as well as the mean of all subjects (marked as Δ). The CON group showed no significant changes, whereas in VIB group the time interval until exhaustion was significantly enhanced.

The grand mean of muscle endurance of the VIB and CON is illustrated in Figure 4. The analysis of variance revealed a significant interaction effect of group×time for local static muscle endurance. For instance, muscle endurance increased significantly more for the VIB than for the CON group. Moreover, a significant main effect for the factors group (P<0.05) and time (P<0.05) were observed, indicating a decreased sway path after the training period for both groups. In the VIB group, the time interval until maximal fatigue was reached was enhanced significantly by 36% after four weeks of WBV treatment. The performance of the CON group showed a non-significant improvement of 11% (p = 0.49).

## Discussion

The main finding of this study is that (1) there was an additional effect of WBV exposure on balance control and local static muscle endurance. However, (2) there was no significant effect on jump height.

In this study, the CON intervention program consisted of exactly the same exercises as those performed by the VIB group in order to evaluate the superimposed effect of vibration training. Interestingly, our results reveal that the performance gains in balance control and local static muscle endurance were significantly higher in the VIB group than the CON group after four weeks of training.

### Balance Control

In numerous studies, vibration training has been shown to improve balance in sub-populations such as elderly people [41], [6] or patients with motor impairments or comprised health suffering from Parkinson [42], [43], stroke [44], [45], or multiple sclerosis [46]. However, the present results provide evidence for an enhancement in balance control in healthy well-trained subjects as well. The COP displacement in anterior-posterior and medio-lateral direction was significantly reduced after the four-week WBV training regimen, whereas the improvements in the CON group remained insignificant. Since the training variables for both groups were leveled, the improvements in balance control most likely can be attributed to the WBV treatment.

The effective mechanisms underlying the improvements in balance control are hardly understood. However, it is well documented that side-alternating WBV training causes rapid vertical and horizontal displacement with particularly high levels of acceleration [47], [48] classified as perturbations [2]. Hence, you could expect that vibration, as a stimulus that interferes with postural control, leads to an immediate deterioration of balance [49], [2]. In general, perturbations are known to be an appropriate training stimulus regarding balance control. After perturbation training, postural control was improved to a similar extend as in response to balance training [50]. Moreover, WBV is associated with a strong inhibition of spinal reflexes. Ia afferent transmission [51]–[54] was highly diminished during and after WBV. Very similar observations have been reported in regards to balance training, which was shown to be accompanied by a strong reduction in reflex activity [39], [55]–[57]. The inhibition of spinal reflexes in balance tasks assumably relies on the prevention of reflex-mediated joint oscillations interfering with postural control and on a transfer in movement control to supraspinal centers [58], [59]. Both aspects are associated with an improvement in balance control [60]. Although to date there is no study investigating long-term adaptations of spinal reflexes due to WBV training, the above-mentioned aspects point towards a similarity in acute neuromuscular modulation regarding balance and WBV training. Thus, highly diminished spinal reflexes and a reduction in spinal excitability in response to WBV might have contributed to the improvement in balance control.

Furthermore, vibration-induced changes in joint stiffness [31], [32], which is accompanied by an improvement in ankle and knee joint stabilization [61], [62], [5], [35], [33], also might have contributed to the improvement in balance control. According to literature it is well known that the general increase in neuromuscular activation in response to WBV leads to an acutely enhanced co-activation of lower extremity extensor and flexor muscles [33], [5]. This co-activation is considered to have a positive effect on joint stiffness [31], [32] associated with altered postural control strategies during WBV [5], [33]. Moreover, there was an association between the improvement in ankle [34] and knee joint stability [35], [36] after four weeks of WBV training and the improvement in neuromuscular control. Based on this relation the enhancement in joint stabilisation [32], [35], [36] could have contributed to the gain in balance performance in response to WBV training in this study.

Despite there being evidence for similarities regarding WBV and balance training that could explain the performance gain in balance control, the results of the present study are contrary to those of Mahieu et al. [4]. They [4] investigated the effect of a four-week WBV training against an equivalent exercise program performed without vibration in competitive skiers and could not find significant changes regarding reactive balance control. A different test setting or the different group of subjects might have caused the equivocal observations. We used a static balance task to assess changes in balance control; on the other hand, Mahieu et al. [4] chose a dynamic balance task which was specifically distinguished by reactive COP displacements - a task competitive skiers might be used to. In addition, although the participants in the present study were well-trained healthy subjects, it might be proposed that skiers are much more used to being exposed to situations that require balance control [63], [64].

Based on this one can conclude that sub-populations such as elderly people or patients with motor impairments as well as well-trained healthy adults could profit from WBV training, at least within vibration-dose dependent limits regarding the duration in regard to elderly people or patients [1], [19], [2]. For athletes familiar with balance tasks due to the specific competitive demand of their discipline, WBV seems to provide no additive benefit.

### Jump Height

This study revealed no changes in jump height in response to WBV training; neither the CON nor the WBV group showed significant differences. Although our results are well in line with the findings of Ronnestaad et al. [13] and de Ruiter et al. [11], the majority of WBV-related articles document an increase in jump power [26] and jump height [65], [3], [66] in response to WBV exposure. These findings are confirmed by a recently published meta-analysis restricted to randomized controlled trials that shows a superimposed effect of vibration training in both knee extension muscle strength and countermovement jump height above identical interventions conducted without WBV [67]. After analyzing the contrary findings between various studies [65], [3], [4], [66], [11], [13], [12] with respect to the specific differences regarding the intervention modalities and vibration settings [1], [14], [67], it was detected that the type of WBV platform, as well as amplitude and frequency considerably determines to which extent muscle strength and muscle power gains are achieved in response to WBV exposure [67]: The combination of high frequencies [65], [3], [66] and low amplitudes [65], [66], [26] are supposed to be particularly beneficial. Furthermore, from a neuromuscular perspective, highly augmented activation intensities of the knee extensors (i.e., the muscles mainly responsible for a vertical jump performance) are associated with vibration training settings performed with a distinct knee flexion instead of extended knee joints [21]. Thus, body position is considered to be another major factor regarding WBV training purposes [21], [19]. Since we selected a high vibration frequency, which is assumed to be beneficial regarding jump performance [3], [65]–[67], it can be suggested that body position and vibration amplitude are the major factors responsible for the lacking intervention effect.

In summary, there is evidence for an additive enhancement in jump performance above conventional exercise in response to WBV training in literature [65], [66], [26,26]. However, at least within our parameter selection WBV training has no additional effects above the overall adaptations due to intervention alone. As a consequence it may be speculated that improvements in jump height, as observed in several other vibration-related studies, can only be achieved when an appropriate WBV setting is chosen.

### Local Static Muscle Endurance

A main outcome of this study is the additional effect of WBV exposure on local muscle endurance. It is well documented that WBV causes acute changes in endurance-associated parameters such as energy metabolism and turnover [15], [27], [37], leg blood flow [16], [17] and neuromuscular activation in the lower extremities [21], [20], [19]. A significant increase in local muscle perfusion in the gastrocnemius and vastus lateralis muscle as well as a 100% increase in popliteal blood flow were found during and after termination of vibration exercise [28]. Moreover, Rittweger et al. [15] observed an increase in VO2, dependent on frequency and amplitude and an augmented energy turnover during WBV training [27]. Accordingly, one would expect an enhanced energy demand arising from exposure to vibration. Those physiological changes are accompanied by changes in neuromuscular activity. EMG activity was shown to be distinctly increased due to WBV in a static and dynamic muscle contraction mode [19], [21]. Such an increase in EMG activity is associated with an increase in muscle fibre recruitment and frequency [68], [69]. Thus, one might argue that metabolic and energetic changes occur in response to an enhancement in activation intensity in the muscles of the lower extremities that are stimulated throughout vibration exposure [48], [21]. Altogether, those observations point towards changes in endurance-associated parameters due to WBV exposure and could explain the improvement in local static muscle endurance in response to WBV training that has been observed in this study. Additionally, based on the study of Stewart et al. [70] it might be suggested that training duration has supplementary influences beyond that. In our present study, duration was rather long (with an average of more than 6 and 13 min in the first and second set, see Table 1). Although there seems to be a knowledge deficit regarding the effect that the time of exposure to vibration has on performance [1], it might be speculated that the longer the muscles are exposed to WBV the higher the neuromuscular and metabolic demand should be [27].

In conclusion, WBV training causes an additional effect on balance control and local static muscle endurance; however, jump performance remains unaffected. It might be speculated that the improvements in local muscle endurance could be caused by adaptations in energy metabolism and turnover, which is associated with vibration-induced changes in neuromuscular activation. The enhancement in balance control might be caused by the perturbation-like stimulus due to rapid mechanical oscillation during WBV. Platform displacements with high acceleration transmitted quickly throughout the entire body might interfere with postural control and cause immediate deterioration, and thus, could provide a training stimulus entailing adaptations in balance control.

However, in our study there is no evidence for a vibration-induced change in jump performance. As vibration amplitude and frequency as well as the extent of knee flexion are known to considerably determine the extent of the vibration-induced muscle strength and muscle power gains, it can be speculated that the training setting in our study was not appropriate to cause sufficiently high activation intensities in the target muscle groups and finally cause adaptations.

In summary, this study provides evidence for an additional effect of WBV beyond conventional exercise alone regarding the parameters associated with balance control and muscle endurance. Thus, a training program that includes WBV can provide supplementary benefits in young well-trained adults compared with an equivalent program that does not include WBV.
